# Supplementary material for: Two RhoGEF isoforms with distinct localisation control furrow position during asymmetric cell division
Source: Nat Commun. 2023 Jun 2;14:3209. doi: 10.1038/s41467-023-38912-9 (PMC10238489; doi:10.1038/s41467-023-38912-9)
Supplement: Supplementary file 1 — Supplementary Information [file 41467_2023_38912_MOESM1_ESM.pdf]

Two RhoGEF isoforms with distinct localisation control furrow position during asymmetric cell division

Emilie Montembault[1,2¶], Irène Deduyer[1,2¶], Marie-Charlotte Claverie[1,2¶], Lou Bouit[1,3], Nicolas J. Tourasse[4], Denis Dupuy[4], Derek M[C]Cusker[1,2] and Anne Royou[1,2*]

[1] CNRS, UMR5095, University of Bordeaux, Institut Européen de Chimie et Biologie, 2 rue Robert Escarpit, 33607 Pessac, FRANCE

[2] Current address, CNRS, UMR5095, University of Bordeaux, Institut de Biologie et Génétique Cellulaire, 1 rue Camille Saint-Saëns, 33077 Bordeaux, FRANCE

[3] Current address: CNRS, UMR5297, University of Bordeaux, FRANCE

[4]University of Bordeaux, INSERM, U1212, Institut Européen de Chimie et Biologie, 2 rue Robert Escarpit, 33607 Pessac, FRANCE

[¶] Contributed equally

* Correspondence:
anne.royou@u-bordeaux.fr
phone: +33 (0)5 56 99 6511

Supplementary informations

Supplementary Tables

Table 1. Number of reads and the corresponding percentage of exon junction usage from RNAseq analysis of the *pbl* gene in various Drosophila tissues.

Tissues	Exon junctions	6-9	6-8	6-7	total	13-15	13-14	total	11-13	11-12	total
	isoform	A, C, E	B, D	F		A, B	E		A, B, **E**	C, D	
Overall	reads	58360	48104	1799	108263	73073	2094	75167	102736	4524	107260
	%	54	44	2	100	97	3	100	96	4	100
embryos	reads	7685	14155	0	21840	12602	1283	13885	19864	638	20502
	%	35	65	0	100	91	9	100	97	3	100
adults	reads	1429	1241	117	2787	2478	8	2486	2981	165	3146
	%	51	45	4	100	99.7	0.3	100	95	5	100
CNS	reads	133	179	0	312	367	0	367	318	6	410
	%	43	57	0	100	100	0	100	98	2	100
Ovaries	reads	2883	2655	0	5538	3440	0	3440	5588	153	5741
	%	54	46	0	100	100	0	100	97	3	100
Testes	reads	43	112	280	435	131	1	132	146	8	154
	%	10	26	64	100	99	1	100	95	5	100

Table 2. Nomenclature and the corresponding genotypes of larvae used for the indicated figures

Name	Genotype	Fig.
WT<br>*pbl*^{MS}	*yw; P[$_{sqh}$>sqh::GFP42]/+ ; +*<br>*yw; P[$_{sqh}$>sqh::GFP42]/+ ; pbl*^{MS}	1, S1<br>1, S1
GFP::Pbl	*yw;; P[$_{pbl}$>GFP::Pbl(68A4)]*	2e
VenusFP::RacGAP50C<br>GFP::Pbl-A<br>GFP::Pbl-B	*yw; P[$_{UAS}$>VenusFP::RacGAP50C]; P[$_{69B}$>Gal4]/+*<br>*yw;; P[$_{pbl}$>GFP::Pbl-A(86F8)]*<br>*yw;; pbl*^{2}*/pbl*^{3}*, P[$_{pbl}$>GFP::Pbl-B(86F8)]*	3<br>3<br>3
Pbl A+B<br>*Pbl-A*<br>*Pbl-B*	*yw; P[$_{sqh}$>sqh::GFP42]/+ ; pbl*^{2}*/pbl*^{3}*, P[$_{pbl}$>Pbl-A+B (86F8)]*<br>*yw; P[$_{sqh}$>sqh::GFP42]/+ ; pbl*^{2}*/pbl*^{3}*, P[$_{pbl}$>Pbl-A (86F8)]*<br>*yw; P[$_{sqh}$>sqh::GFP42]/+ ; pbl*^{2}*/pbl*^{3}*, P[$_{pbl}$>Pbl-B (86F8)]*	4<br>4<br>4
Pbl A+B<br>*Pbl-A*<br>*Pbl-B*	*yw;; pbl*^{2}*/pbl*^{3}*, P[$_{pbl}$>Pbl-A+B (86F8)]*<br>*yw;; pbl*^{2}*/pbl*^{3}*, P[$_{pbl}$>Pbl-A (86F8)]*<br>*yw;; pbl*^{2}*/pbl*^{3}*, P[$_{pbl}$>Pbl-B (86F8)]*	S5<br>S5<br>S5

Pbl-B	*yw; P[$_{sqh}$>sqh::GFP42]/p[$_{h2az}$>H2A.Z::mRFP] ; pbl^{2}/pbl^{3}, P[$_{pbl}$>Pbl-B (86F8)]*	S6
RacGAP50C RNAi GFP::Pbl-A	*yw; P[$_{UAS}$>RacGAP50C dsRNA]/+ ; P[$_{69B}$>Gal4]/ P[$_{pbl}$>GFP::Pbl-A(86F8)]*	5a
RacGAP50C RNAi GFP::Pbl-B	*yw; P[$_{UAS}$>RacGAP50C dsRNA]/+ ; P[$_{69B}$>Gal4]/ P[$_{pbl}$>GFP::Pbl-B(86F8)]*	5a
Control	*yw; P[$_{sqh}$>sqh::GFP42]/+ ; P[$_{69B}$>Gal4]/+*	5b, S7
RacGAP50C RNAi	*yw; P[$_{UAS}$>RacGAP50C dsRNA]/ P[$_{sqh}$>sqh::GFP42] ; P[$_{69B}$>Gal4]/+*	5b, S7

Table 3. Nomenclature and the corresponding sequence of primers mentioned in the Methods section

Primer name	Sequence	**Use**
Forward pair A<br>Reverse pair A	CGCGTATTTCTTGGCGCCCCC<br>CATTCCTTTTGCTTGTGTATCGCCTCGAAG	Amplification fragment A of genomic pbl: 4833nt
Forward pair B<br>Reverse pair B	TGGCAGTTGTGTTTGTTTAGTTGCCCGG<br>CATTGCCATGGGCTCTTCTATGGGC	Amplification fragment B of genomic pbl: 3504nt
Forward pair C<br>Reverse pair C	CACACACACACACACTCTGGTTATAGACTTT<br>CATGCTTTGAACGCCTTACGACTTAGGG	Amplification fragment C of genomic pbl: 3328nt
Forward qPCR A form<br>Reverse qPCR A form	CACGCAAGAGAATTTCACCA<br>GTGCTCGTCAACCACGACAT	Amplification of 178nt fragment of pbl-A cDNA for qPCR (efficiency 116,5%, R^2=0,999)
Forward qPCR B form<br>Reverse qPCR B form	TCTTTGGATTCCCTGTGGAG<br>TCGGGTGGGTTAATTAGGGT	Amplification of 184nt fragment of pbl-B cDNA for qPCR (efficiency 88,14%, R^2=0,986)
Forward qPCR actin<br>Reverse qPCR actin	GCGTCGGTCAATTCAATCTT<br>AAGCTGCAACCTCTTCGTCA	Amplification of 138nt fragment of actin cDNA for qPCR (efficiency 100,3%, R^2=0,998)

Supplementary Figures

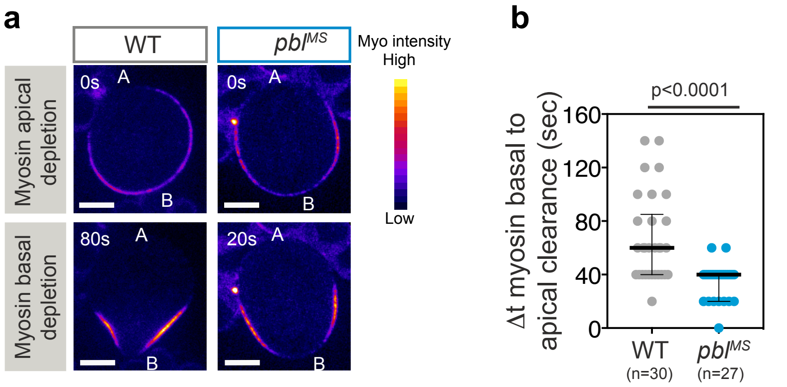

Supplementary figure 1 | The time elapsed between myosin apical and basal depletion is longer in wild-type than *pbl*^{MS} neuroblasts.

(a) Images of wild-type and pblMS neuroblasts expressing Sqh::GFP (myosin) at onset of myosin depletion from the apical (A) and basal (B) poles (top and bottom rows respectively). Time, seconds (s). Scale bars, 5 µm. **(b)** Scatter dot plot showing the time elapsed between myosin apical and basal pole depletion for the indicated genotype. Bars represent median ± interquartile range. A two-tailed Mann-Whitney test was used to calculate the P value.

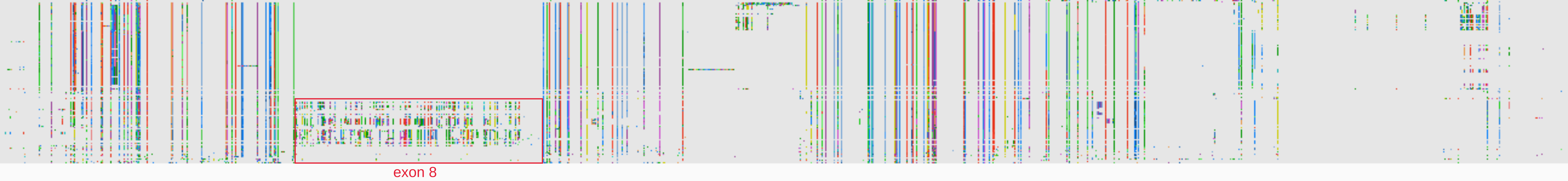
exon 8

Supplementary figure 2 | Multiple alignment of 948 orthologous Pbl protein sequences from eukaryotes.

The figure shows an overview of the alignment of 948 orthologous Pbl proteins sequences. The region corresponding to exon 8 in *D. melanogaster* Pbl-B isoform is boxed in red. This region is covered in 233 out of 948 sequences, mostly from insects.

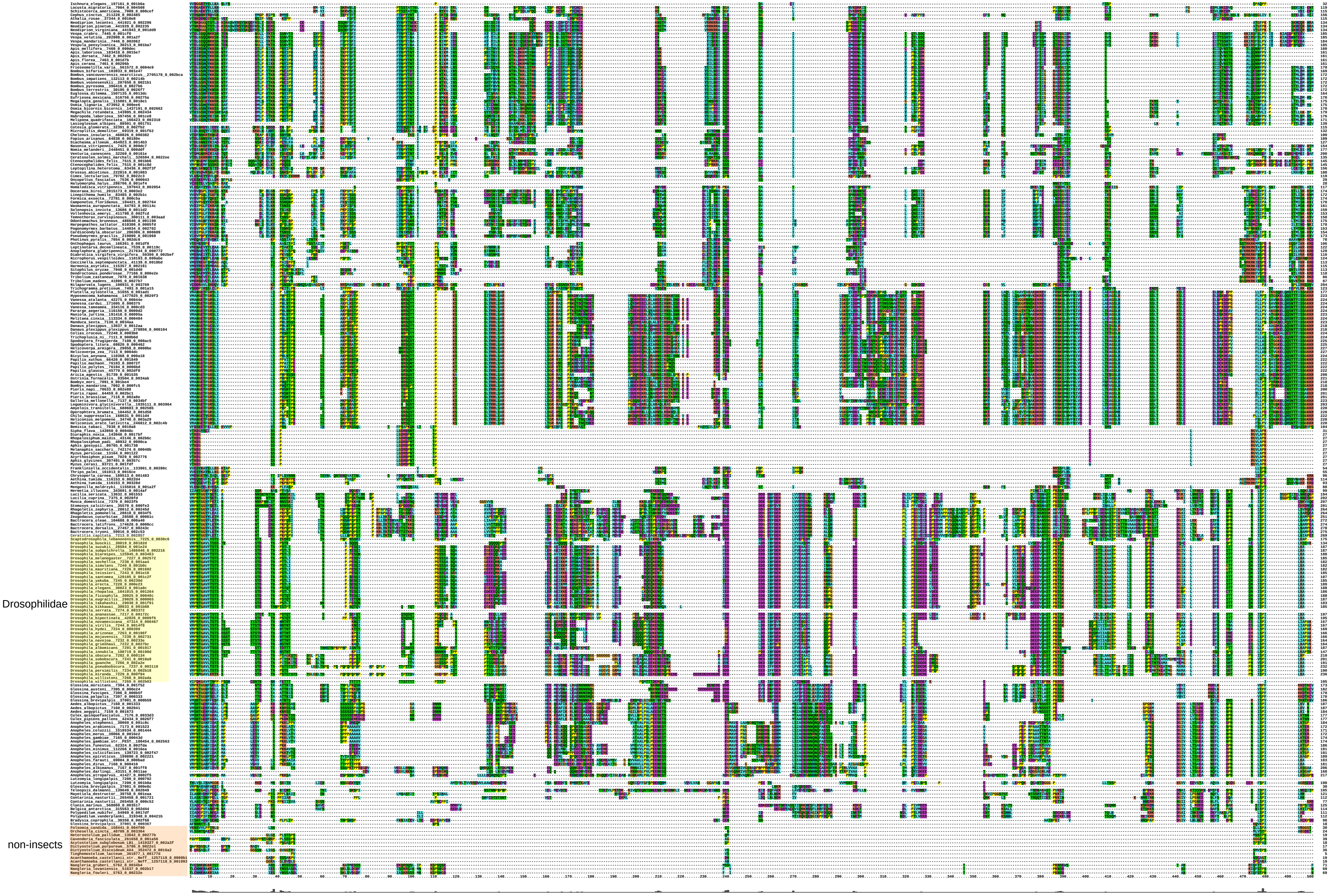
Drosophilidae
non-insects

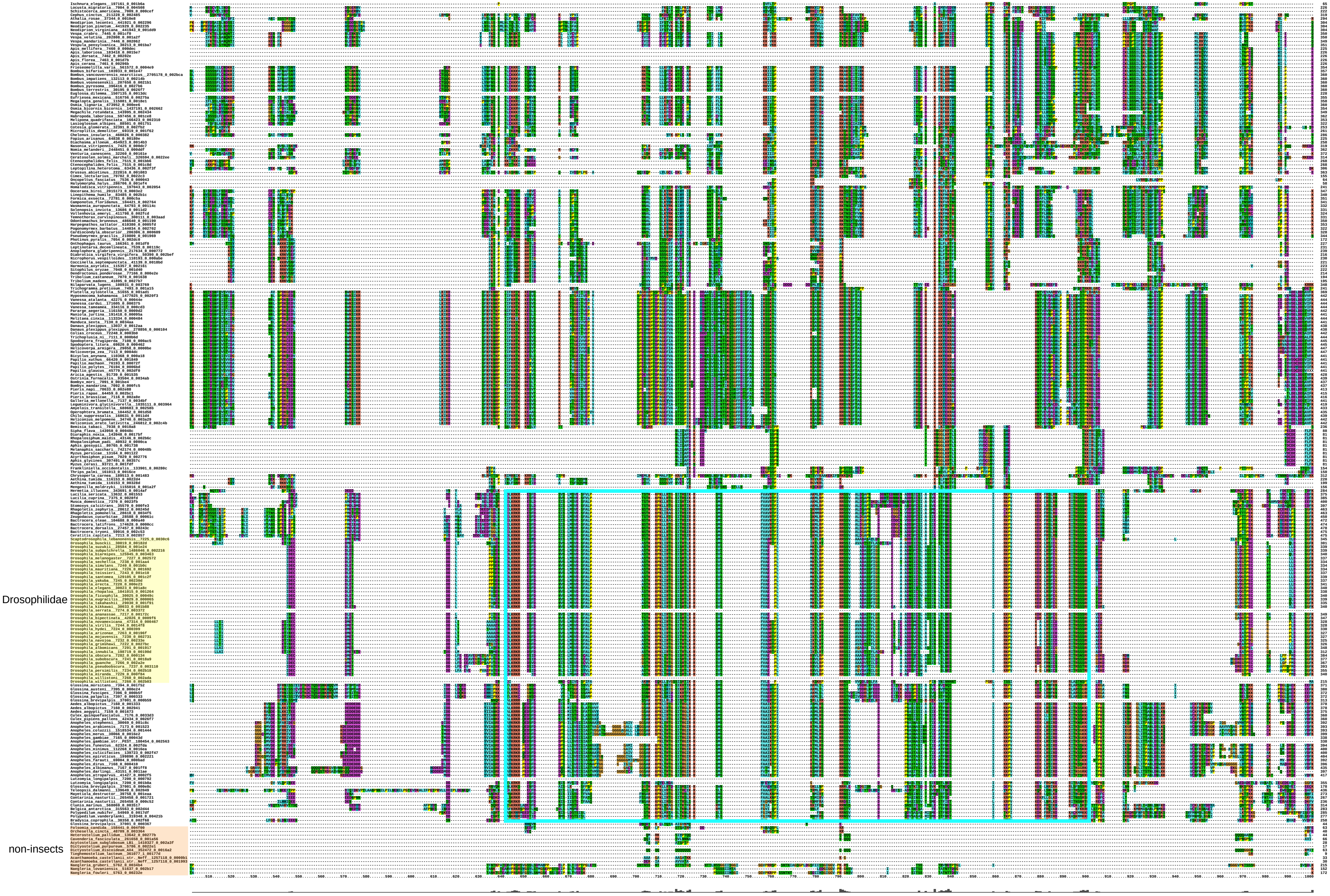
Drosophilidae
non-insects

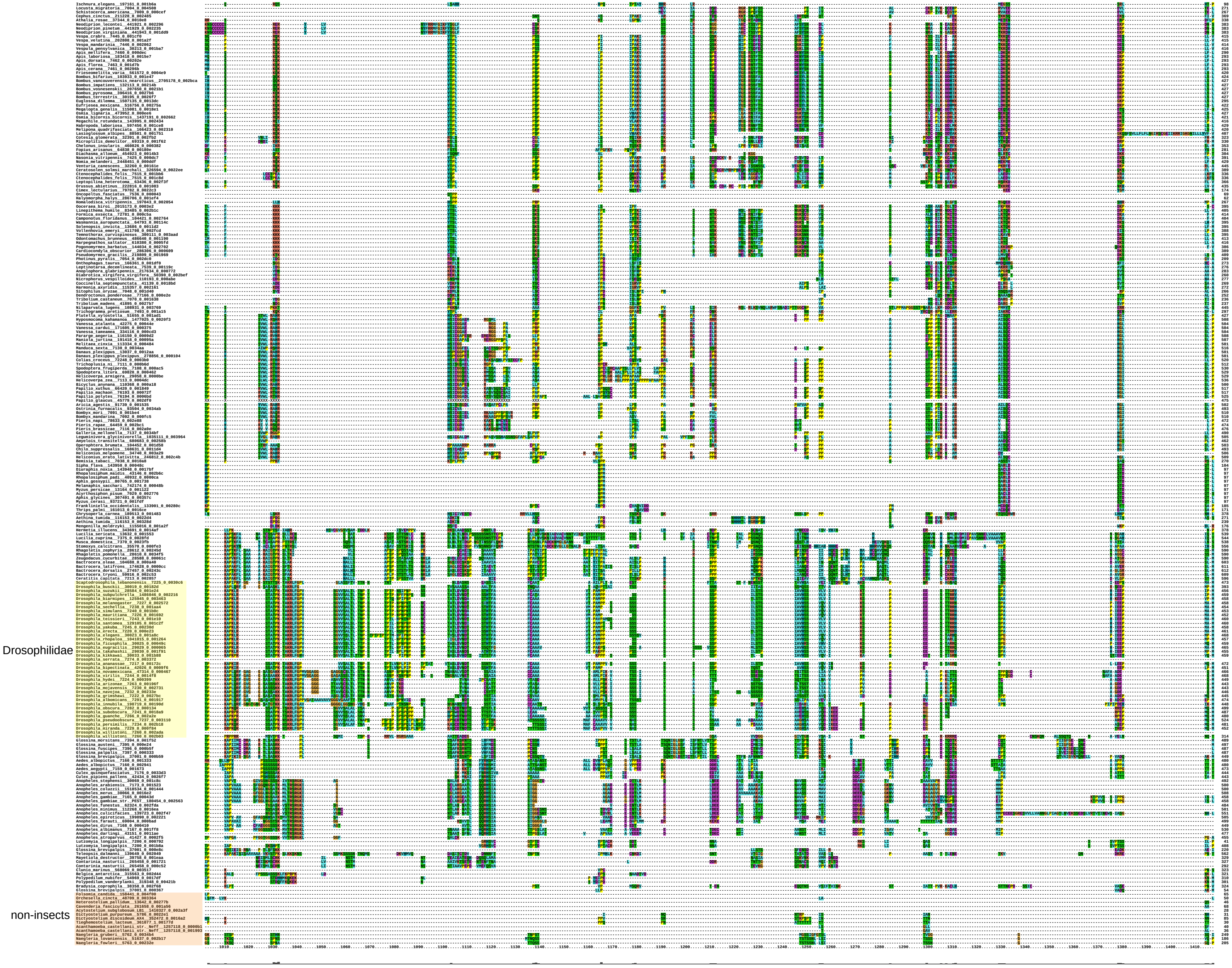
Drosophilidae
non-insects

Supplementary figure 3 | Subset of the multiple alignment of Pbl orthologs showing 233 protein sequences spanning exon 8 of the *D. melanogaster* Pbl-B.

The sequences shown correspond to those included in the red box in fig. S2. All sequences are from insects, with the exception of the 13 sequences displayed at the bottom of the alignment (highlighted in orange). Different sequences are found in various subgroups of insect species. The cyan box highlights a homologous region shared between Drosophilidae (highlighted in yellow) and a subset of other insects including mosquitoes (e.g. *Aedes Culex, Anopheles*). Dark grey bars underneath the alignment indicate sequence conservation. OrthoDB identifiers are given next to species names.

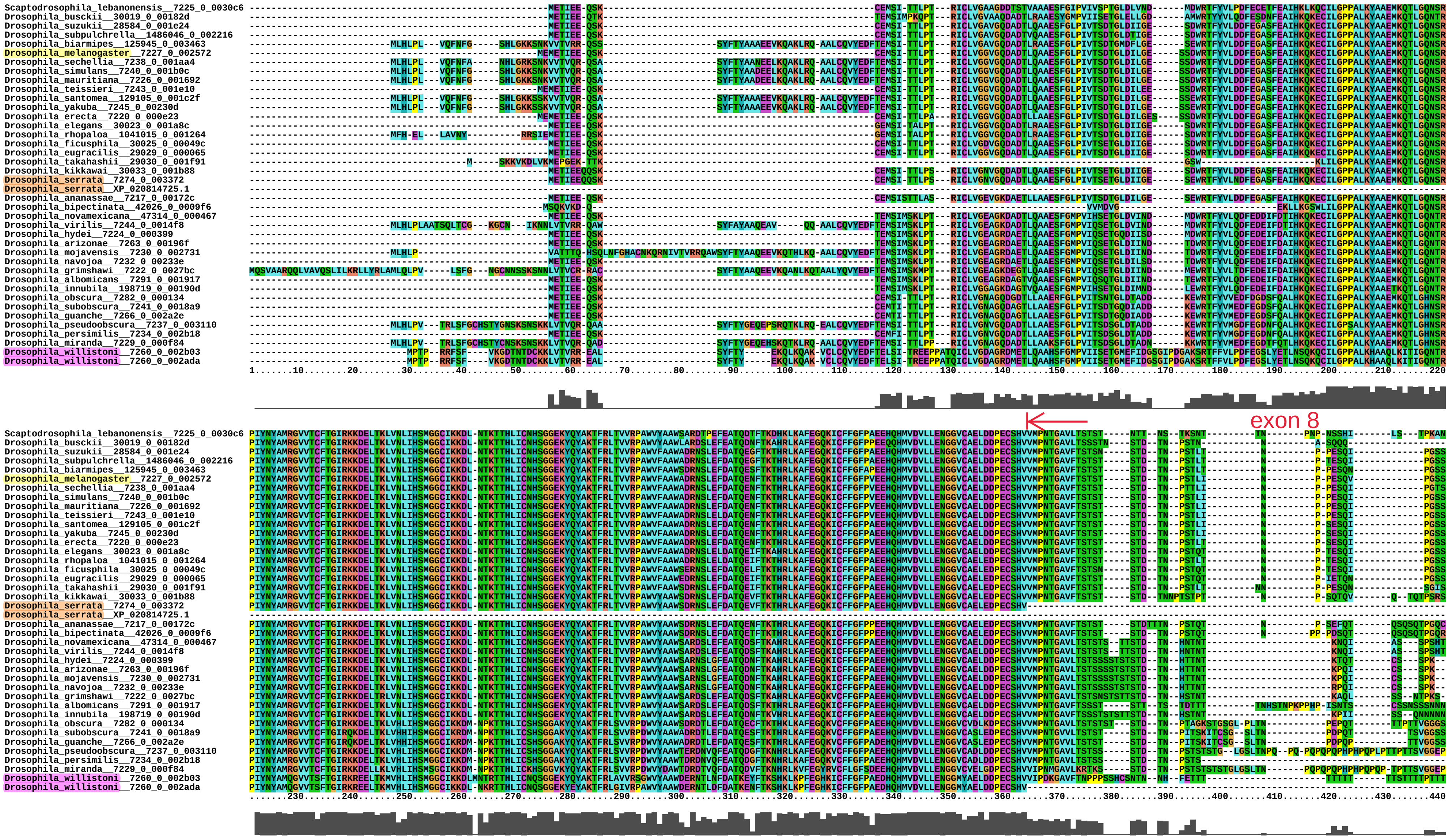
exon 8

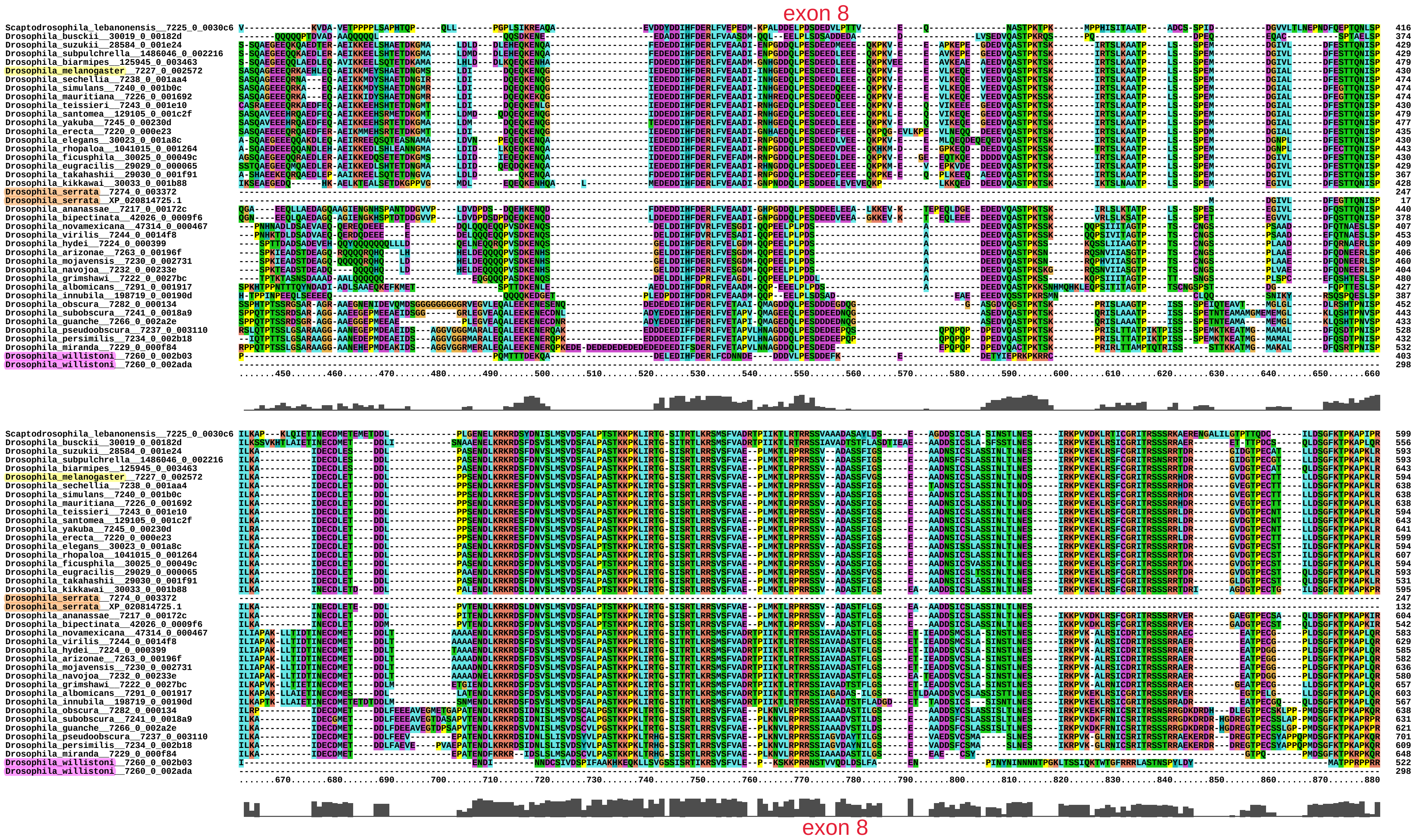
exon 8
exon 8

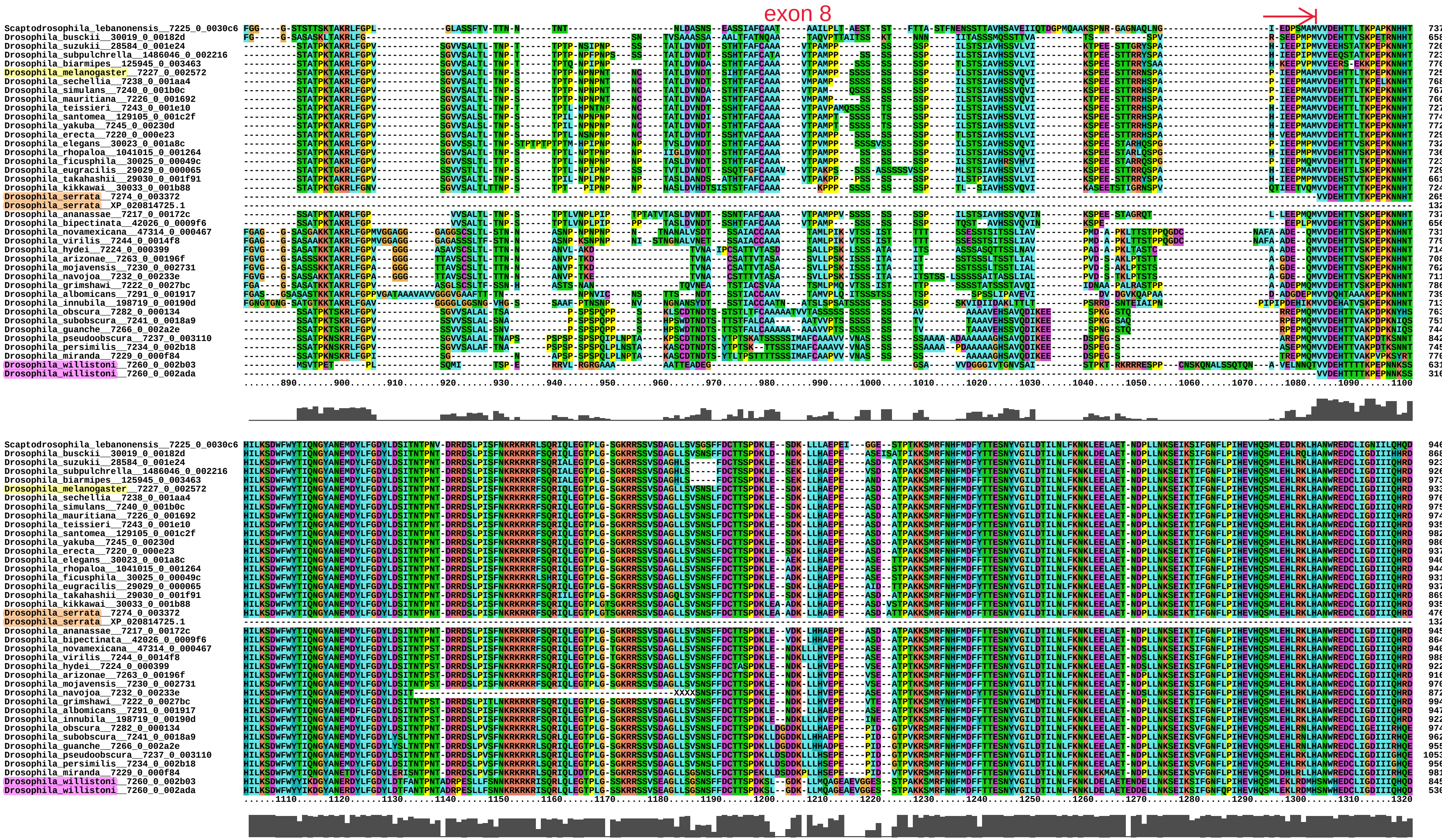
exon 8

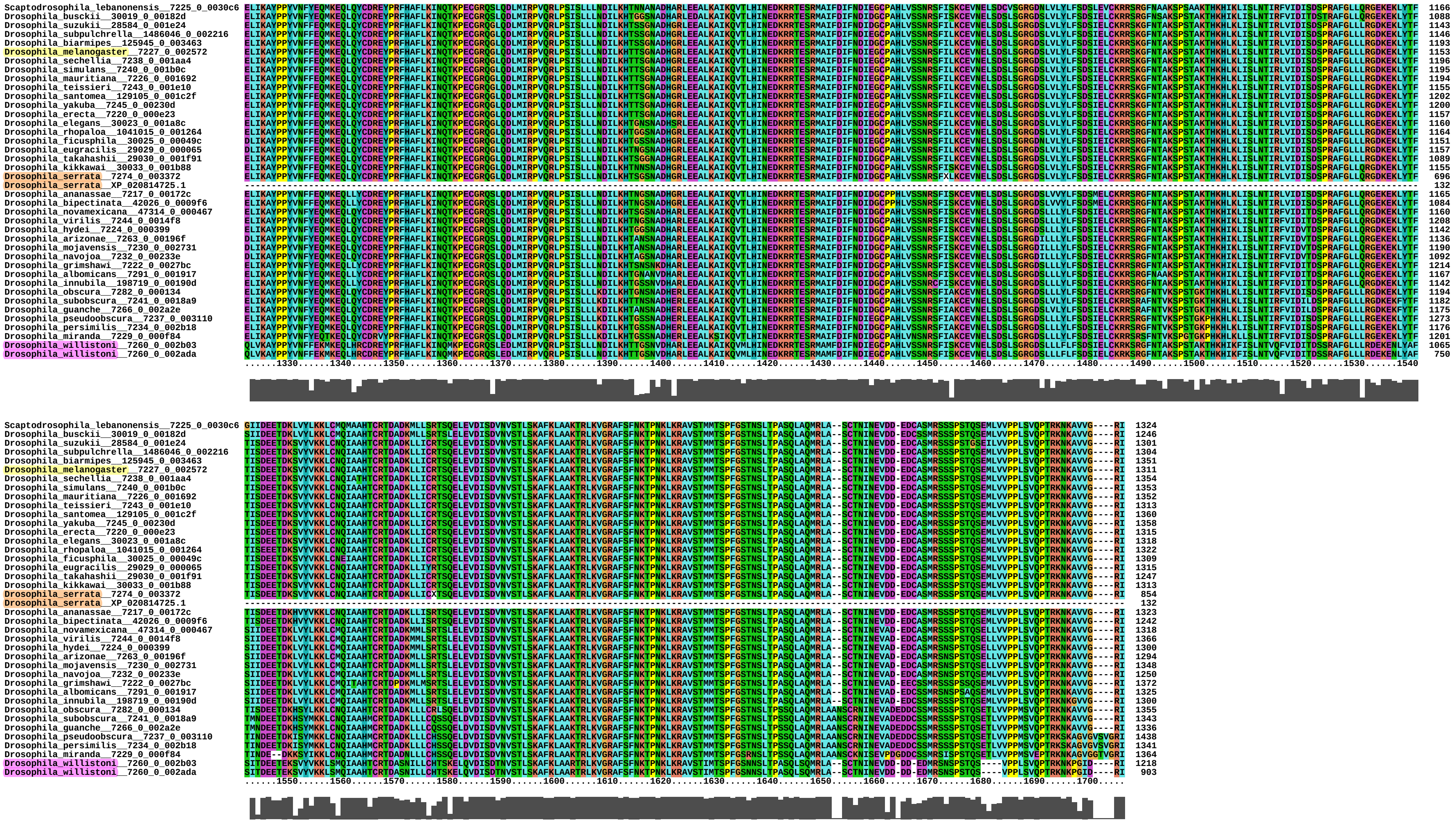
Scaptodrosophila_lebanonensis__7225_0_0030c6
Drosophila_busckii__30019_0_00182d
Drosophila_suzukii__28584_0_001e24
Drosophila_subpulchrella__1486046_0_002216
Drosophila_biarmipes__125945_0_003463
Drosophila_melanogaster__7227_0_002572
Drosophila_sechellia__7238_0_001aa4
Drosophila_simulans__7240_0_001b0c
Drosophila_mauritiana__7226_0_001692
Drosophila_teissieri__7243_0_001e10
Drosophila_santomea__129105_0_001c2f
Drosophila_yakuba__7245_0_00230d
Drosophila_erecta__7220_0_000e23
Drosophila_elegans__30023_0_001a8c
Drosophila_rhopaloa__1041015_0_001264
Drosophila_ficusphila__30025_0_00049c
Drosophila_eugracilis__29029_0_000065
Drosophila_takahashii__29030_0_001f91
Drosophila_kikkawai__30033_0_001b88
Drosophila_serrata__7274_0_003372
Drosophila_serrata__XP_020814725.1
Drosophila_ananassae__7217_0_00172c
Drosophila_bipectinata__42026_0_0009f6
Drosophila_novamexicana__47314_0_000467
Drosophila_virilis__7244_0_0014f8
Drosophila_hydei__7224_0_000399
Drosophila_arizonae__7263_0_00196f
Drosophila_mojavensis__7230_0_002731
Drosophila_navojoa__7232_0_00233e
Drosophila_grimshawi__7222_0_0027bc
Drosophila_albomicans__7291_0_001917
Drosophila_innubila__198719_0_00190d
Drosophila_obscura__7282_0_000134
Drosophila_subobscura__7241_0_0018a9
Drosophila_guanche__7266_0_002a2e
Drosophila_pseudoobscura__7237_0_003110
Drosophila_persimilis__7234_0_002b18
Drosophila_miranda__7229_0_000f84
Drosophila_willistoni__7260_0_002b03
Drosophila_willistoni__7260_0_002ada

Supplementary figure 4 | Subset of the multiple alignments of Pbl orthologs showing protein sequences from 38 Drosophilidae species.

The *D. melanogaster* Pbl-B sequence is highlighted in yellow for reference and the region corresponding to exon 8 is indicated by red arrows and vertical bars above the alignment. Exon 8 is missing in *D. serrata* protein ortholog (identifier 7274_0_003372; highlighted in orange), however it is present at the DNA level, but highly mutated. Two Pbl co-orthologs are present in *D. willistoni* (highlighted in magenta). In one of them (identifier 7260_0_002ada), exon 8 is truly missing from the DNA and protein sequence, whereas the other (identifier 7260_0_002b03) contains a different exon 8 sequence. Dark grey bars underneath the alignment indicate sequence conservation. OrthoDB identifiers are given next to species names.

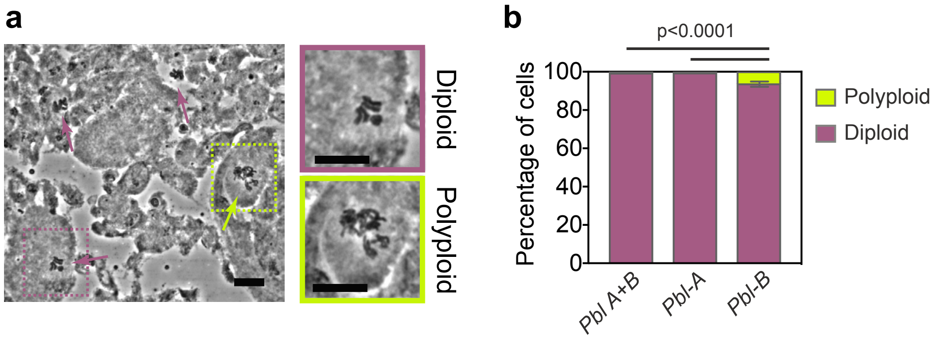

Supplementary figure 5 | Mild increase in the frequency of polyploid cells in the central nervous systems of third instar larvae expressing solely the *Pbl-B* isoform.

(a) Phase contrast image of a squashed larval central nervous system stained with orcein. The purple and yellow arrows point to diploid and polyploidy cells respectively. The insets are magnification of diploid and polyploid cells. Scale bars, 10 µm. **(b)** Frequency of polyploid mitotic cells for the indicated genotype. More than 140 cells were counted per brain and 3 to 4 brains were counted per experiment and per genotype. Three independent experiments were performed. The bars represent the mean±SD. A Chi-Square test was used to calculate P values.

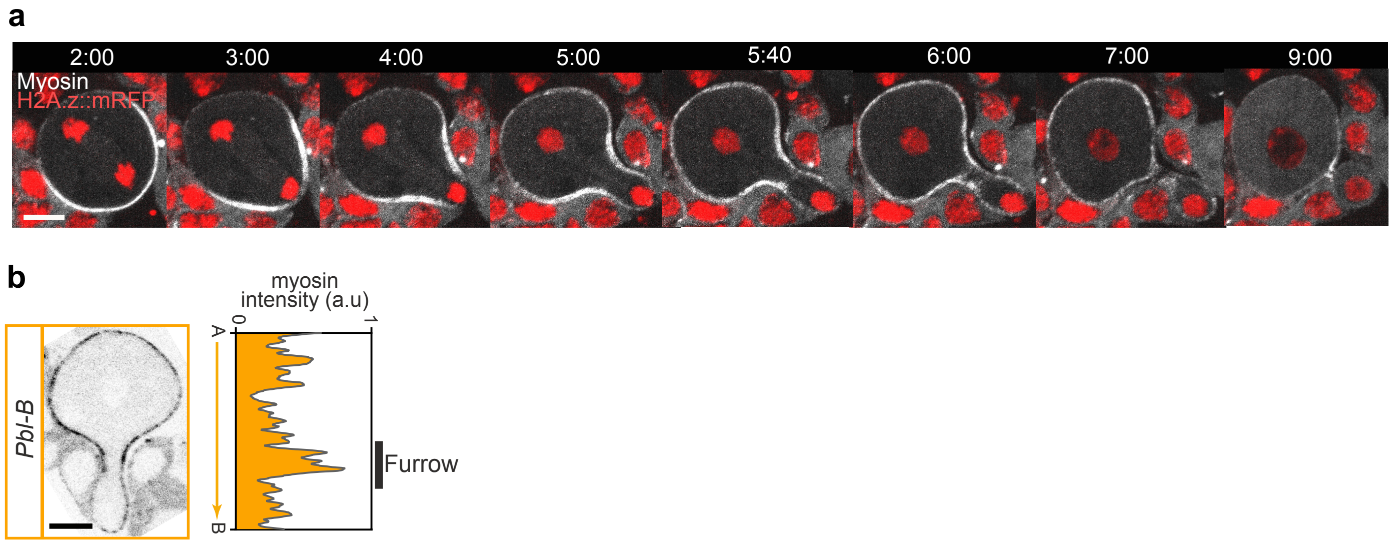

Supplementary figure 6 | No trailing chromatid is observed in Pbl-B expressing cells.
(**a)** Time-lapse images of Pbl-B expressing cells labelled with H2A.z::mRFP (red) and Sqh::GFP (Myosin, gray). Time starts at anaphase onset. Time, min:sec. Scale bar, 5 µm. **(b)** Sagittal image of a neuroblast expressing Pbl-B and Sqh::GFP and the corresponding myosin intensity measurement at the cortex from apical (A) to basal (B) poles. The black vertical line represents the position and width of the furrow on the line-scan. Scale bar, 5µm. (number of cells, n=8).

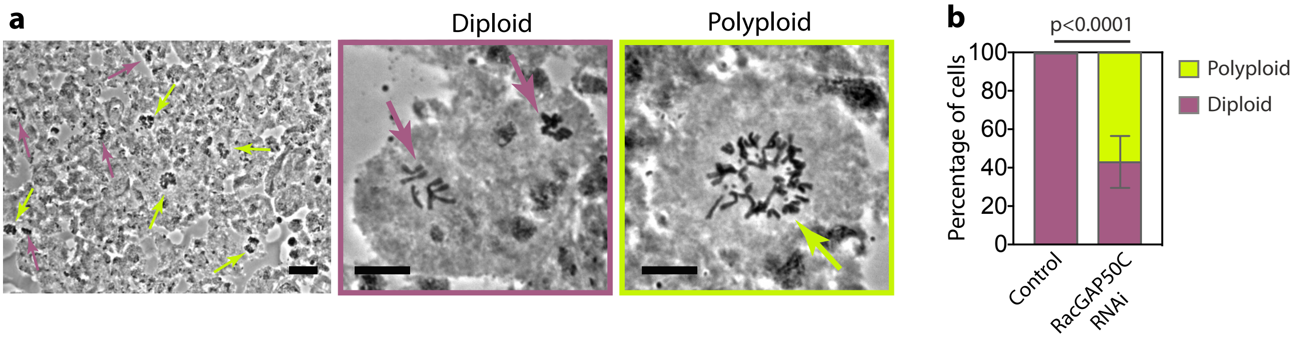

Supplementary figure 7 | Dramatic increase in the frequency of polyploid cells upon RacGAP50C depletion in the third instar larvae central nervous system.
(a) Phase contrast image of a squashed larval central nervous system stained with orcein. The purple and yellow arrows indicate diploid and polyploid cells respectively. The insets are magnification of diploid and polyploid cells. Scale bar, 10µm. **(b)** Frequency of polyploid mitotic cells for the indicated genotype. More than 80 cells were counted per brain. 4 and 10 brains were counted for control (69B>Gal4) and RacGAP50C RNAi (69B>Gal4; P[UAS>RacGAP50C dsRNA]) respectively, in two independent experiments. The bars represent the mean±SD. A Chi-Square test was used to calculate P values.
