# Supplementary material for: Two RhoGEF isoforms with distinct localisation control furrow position during asymmetric cell division
Source: Nat Commun. 2023 Jun 2;14:3209. doi: 10.1038/s41467-023-38912-9 (PMC10238489; doi:10.1038/s41467-023-38912-9)
Supplement: Supplementary file 3 — Description of Additional Supplementary Files [file 41467_2023_38912_MOESM3_ESM.pdf]

Description of Additional Supplementary Files

Supplementary Movie 1. Myosin dynamics during cytokinesis in wild-type and *pbl*^{MS} neuroblasts.

Time-lapse video of wild-type (left) and *pbl*^{MS} (right) neuroblasts expressing Sqh::GFP. Images are maximum projections. Time, min:sec. Time starts at anaphase onset (initiation of sister chromatid separation). Scale bar, 5 µm. The movie corresponds to fig. 1a.

Supplementary Movie 2. RacGAP50C, Pbl-A and Pbl-B dynamics during cytokinesis.

Time-lapse video of neuroblasts expressing VenusFP::RacGAP50C (Left), GFP::Pbl-A (middle) and GFP::Pbl-B (Right) from anaphase onset. The images are maximum projections. Time=min:sec. Scale bar, 5µm. The movie corresponds to fig. 3a.

Supplementary Movie 3. Myosin dynamics during cytokinesis in Pbl-A+B, Pbl-A or Pbl-B expressing cells.

Time-lapse video of *pbl*^{3}/*pbl*^{2} neuroblasts expressing Pbl-A and B, Pbl-A or Pbl-B and Sqh::GFP. Images are maximum projections. Time, min:sec. Time starts at anaphase onset. Scale bar, 5 µm. The movie corresponds to fig. 4c.

Supplementary Movie 4. Myosin dynamics during cytokinesis in control neuroblasts and neuroblasts depleted for RacGAP50C.

Time-lapse video of 69B>Gal4 neuroblasts (left) and RacGAP50C RNAi; 69B>Gal4 (Right) neuroblasts expressing Sqh::GFP. Images are maximum projections. Time, min:sec. Time starts at anaphase onset. Scale bar, 5 µm. The movie corresponds to fig. 5b.
