# Supplementary figures and images for: Two RhoGEF isoforms with distinct localisation control furrow position during asymmetric cell division
Source: Nat Commun. 2023 Jun 2;14:3209. doi: 10.1038/s41467-023-38912-9 (PMC10238489; doi:10.1038/s41467-023-38912-9)

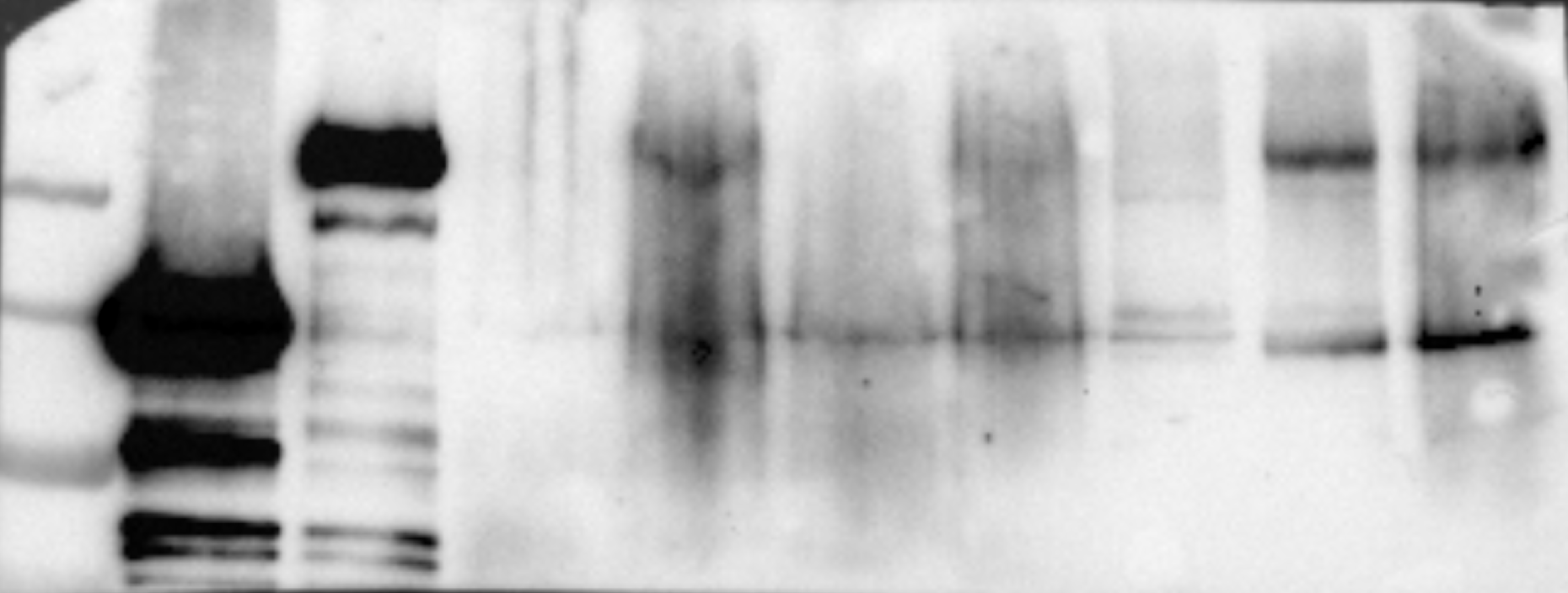

Supplement: Supplementary file 9 — Source Data [file 41467_2023_38912_MOESM9_ESM.zip › SD2.tif]

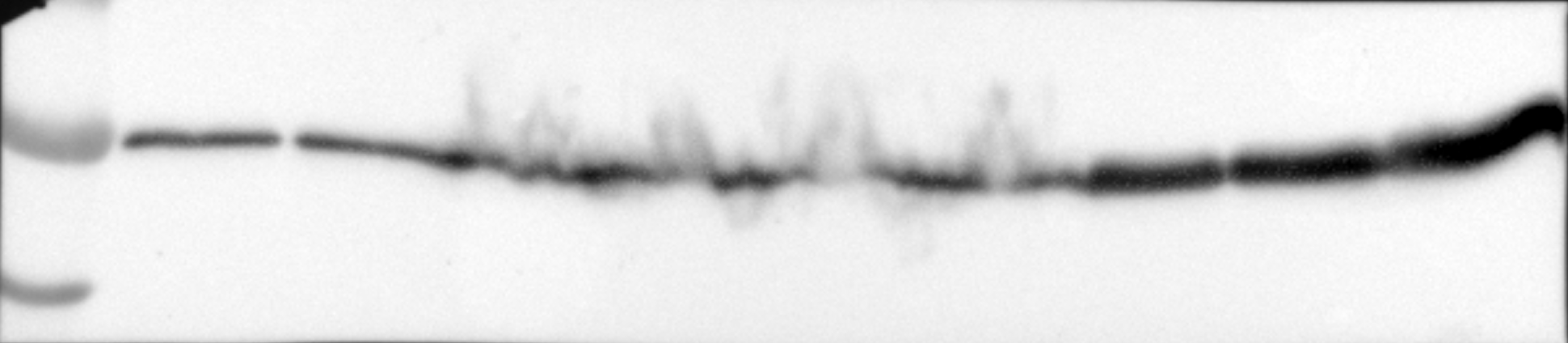

Supplement: Supplementary file 9 — Source Data [file 41467_2023_38912_MOESM9_ESM.zip › SD3.tif]
